# Comparison of pathological clotting using haematological, functional and morphological investigations in HIV-positive and HIV-negative patients with deep vein thrombosis

**DOI:** 10.1186/s12977-020-00523-3

**Published:** 2020-06-22

**Authors:** Brandon S. Jackson, Julien Nunes Goncalves, Etheresia Pretorius

**Affiliations:** 1 Department of Surgery, University of Pretoria, Pretoria, 0007 South Africa; 2 Department of Physiology, University of Pretoria, Pretoria, 0007 South Africa; 3 Department of Physiological Sciences, Stellenbosch University, Stellenbosch, Private Bag X1 Matieland, 7602 South Africa

**Keywords:** Hypercoagulability, Human immunodeficiency virus (HIV), Deep vein thrombosis (DVT), Inflammation

## Abstract

**Background:**

Patients infected with the human immunodeficiency virus (HIV) are more prone to systemic inflammation and pathological clotting, and many may develop deep vein thrombosis (DVT) as a result of this dysregulated inflammatory profile. Coagulation tests are not routinely performed unless there is a specific reason.

**Methods:**

We recruited ten healthy control subjects, 35 HIV negative patients with deep vein thrombosis (HIV negative-DVT), and 13 HIV patients with DVT (HIV positive-DVT) on the primary antiretroviral therapy (ARV) regimen-emtricitabine, tenofovir and efavirenz. Serum inflammatory markers, haematological results, viscoelastic properties using thromboelastography (TEG) and scanning electron microscopy (SEM) of whole blood (WB) were used to compare the groups.

**Results:**

The DVT patients (HIV positive and HIV negative) had raised inflammatory markers. The HIV positive-DVT group had anaemia in keeping with anaemia of chronic disorders. DVT patients had a hypercoagulable profile on the TEG but no significant difference between HIV negative-DVT and HIV positive-DVT groups. The TEG analysis compared well and supported our ultrastructural results. Scanning electron microscopy of DVT patient’s red blood cells (RBCs) and platelets demonstrated inflammatory changes including abnormal cell shapes, irregular membranes and microparticle formation. All the ultrastructural changes were more prominent in the HIV positive-DVT patients.

**Conclusions:**

Although there were trends that HIV-positive patients were more hypercoagulable on functional tests (viscoelastic profile) compared to HIV-negative patients, there were no significant differences between the 2 groups. The sample size was, however, small in number. Morphologically there were inflammatory changes in patients with DVT. These ultrastructural changes, specifically with regard to platelets, appear more pronounced in HIV-positive patients which may contribute to increased risk for hypercoagulability and deep vein thrombosis.

## Clinical implications

- HIV-positive patients do have a hypercoagulable profile compared to HIV-negative patients.
- Increased inflammation is present in patients with DVT.
- Ultrastructural analysis, using the scanning electron microscope, allows a more detailed coagulation profile, especially in HIV-positive patients.

## Background

The prevalence of HIV and Acquired Immune Deficiency Syndrome (AIDS) is a world-wide pandemic. The Joint United Nations Programme on HIV/AIDS (UNAIDS) estimates that 1 million AIDS-related deaths occurred during 2016 [1] and 1.7 million (1.4 million–2.3 million) people were newly infected with HIV by the end of 2018 (http://www.unaids.org/en/resources/fact-sheet). Although treatment of the infection with antiretroviral regimes (ARVs) is essential to addressing the pandemic, the condition is characterized by a large plethora of additional conditions associated, and also co-existing with the HIV infection, including the presence of systemic inflammation. Systemic inflammation is associated with an increase in circulating pro-inflammatory biomarkers, and is closely associated with an amplified propensity to form pathological blood clots (which is associated with hypercoagulability or an over-activated coagulation system) [2–8].

During HIV infection, various circulating inflammatory biomarkers, including cytokines interleukin (IL)-1β, IL-2, IL-6, IL-8, IL-10, IL-12p70, tumor necrosis factor (TNF)-α and also other pro-inflammatory biomarkers are present [9]. An increase in these biomarkers are also present in cardiovascular disease [10, 11] and it is therefore not surprizing that HIV positive individuals are known to have an increased presence of cardiovascular complications [12, 13], including an increased risk to develop atherosclerosis and venous thromboembolic disease [14] and also DVT [15–17]. The presence of DVT is also classified as a systemic inflammatory process [18], and associated with pathological clotting and upregulated circulating inflammatory biomarkers [19].

The prevalence of developing a DVT in HIV positive individuals is increased two to ten times compared to the general population [20]. HIV positive individuals also have a 43% increase in age-adjusted odds ratio for pulmonary embolism, a common complication of DVT, compared to HIV negative individuals [16]. Multiple coagulation abnormalities have been reported in HIV positive patients such as decreased levels of protein C and S; and increased levels of von Willebrand factor [21–23]. However, the association of these abnormalities with DVT is not always consistent [21, 24]. Coagulation investigations are therefore not performed routinely in patients with HIV infection. Standard coagulation investigations are also not performed routinely as part of the management in patients with DVT, with the exception of a D-dimer which is used to assist with the diagnosis [25–27].

In the current paper, we therefore study the haematological profiles, including clotting and various inflammatory markers, in the presence of DVT in HIV positive and HIV negative individuals and compare the results to that of healthy individuals. We compare inflammatory markers for iron (iron saturation, transferrin and serum ferritin), fibrinogen, high-sensitive C-reactive protein (CRP), erythrocyte sedimentation rate (ESR) and haematology analyser results, together with viscoelastic properties of whole blood (WB) and platelet poor plasma (PPP). We also looked at ultrastructure of platelets and erythrocytes/red blood cells (RBCs) (using whole blood smears) with the SEM, as well as after thrombin was added to whole blood, to study clot structure.

## Materials and methods

The aim was to compare the inflammatory and haematological profile of HIV patients with DVT to HIV negative patients with DVT. An analytical and descriptive prospective case control study was used from 2 Pretoria academic hospitals, Kalafong Provincial Tertiary and Steve Biko Academic Hospital, both from urban South Africa. Ten healthy control subjects, 35 HIV negative patients with DVT (HIV negative-DVT), and 13 HIV patients with DVT (HIV positive-DVT) on the primary ARV regimen-emtricitabine, tenofovir and efavirenz—were recruited for the study. For each individual, five blood tubes of venous blood were drawn (this included ethylenediamine tetraacetic acid, buffered tri-sodium citrate and serum separator tubes). The Research Ethic Committee, Faculty of Health Sciences, University of Pretoria, South Africa approved the study (Ethics reference number: 547/2015). Inclusion criteria for the healthy individuals were known HIV negative status and no medical history of any chronic diseases. Patients were included in the research groups if they had confirmed HIV status and confirmed symptomatic DVT on doppler ultrasound or comparative imaging, such as a venogram or computerised tomography scan. In order to minimise opportunistic infections as a confounding factor, HIV positive patients were only included with a World Health Organization Clinical stage 1 and CD4+ cells greater than 170 absolute number (per mm^3^). Exclusion criteria for the healthy individuals and for DVT patients include smoking, pregnancy‚ or the use of any inflammatory-, anticoagulant-, antiplatelet-, hormone replacement- or oral contraceptive- medication.

### Inflammatory marker analysis

Serum iron (total iron in blood) levels were measured together with iron saturation, transferrin (iron binding protein) and serum ferritin (iron storage form). Serum iron levels were measured by a modification of the automated AAII-25 colorimetric method. Fibrinogen (quantitative measurement of functional fibrinogen by automated coagulation analysers), CRP (measured by latex-enhanced nephelometry) and ESR (measured by an automated ESR analyser) levels were also assessed.

### Haematology analysis

A haematology analyser (Advia 2120i, Siemens Healthcare) was used to do full blood counts, and the analysis included white cell count (and its differential count), RBC count, haemoglobin level, haematocrit, mean corpuscular volume (MCV), mean corpuscular haemoglobin (MCH), the mean corpuscular haemoglobin concentration (MCHC), as well as platelet count and mean platelet volume (MPV).

### Viscoelastic properties of whole blood and platelet poor plasma using thromboelastography

Citrated WB, as well as PPP were used. Whole blood, collected in a citrate tube, was centrifuged to obtain PPP (15 min at 3000*g*). Whole blood was used to assess the full coagulation process, while PPP was used to assess coagulation without the influence of platelets and RBCs on the viscoelastic properties of the clot. Calcium chloride was added to either WB or PPP and 7 different parameters measured, which included: reaction time (R-time), kinetic time (K-time), alpha angle, maximum amplitude (MA), maximum rate of thrombus generation (MRTG), time to maximum rate of thrombus generation (TMRTG) and total thrombus generation (TTG).

### Ultrastructure of platelets and red blood cells (RBCs)

The ultrastructure of platelets and RBCs were studied after preparing whole blood smears for scanning electron microscopy (SEM). 10 µl of WB was placed directly on a glass microscope slide, followed by fixing in 2.5% glutaraldehyde, dehydration (as per usual SEM preparation) [28] and mounting. Micrographs were taken with Zeiss Crossbeam 540 Field Emission Gun Scanning Electron Microscopy.

### Statistical analysis

Statistical analyses were performed on GraphPad Prism 5. All data were subjected to one-way ANOVA analysis. A post-test to compare groups was performed using Tukey’s multiple comparison test. Values of significance stated at *P *< 0.05.

## Results

Table 1 demonstrates the demographics of the study.

**Table 1:** Demographics

Groups	N	Male	Female	Age-mean (range)	CD4 count-mean (range)
Controls	10	6	4	30 (26–32)	N/A
HIV negative-DVT	35	11	24	51(19–81)	N/A
HIV positive-DVT	13	2	11	41 (24–69)	511 (178–1764)

*N/A* not applicable

### Inflammatory marker and haematological parameter analysis

Inflammatory marker analyses are shown in Table 2 and haematology analysis are shown in Table 3. Markers with no available results were excluded. The HIV negative-DVT group appeared to have anaemia when compared to the control group, but when adjusted for gender the HIV negative-DVT group still had haemoglobin mean values within the normal reference ranges for male and females, respectively‚ and only the females had decreased serum iron, transferrin, and ferritin levels. The HIV positive-DVT group had anaemia and‚ when adjusted for gender‚ demonstrated low haemoglobin levels for both males and females (furthermore the females also had decreased red cell count, haematocrit, mean cell volume and mean cell haemoglobin concentration), as well as decreased levels of serum iron and transferrin in both genders. The changes in serum iron, transferrin and ferritin in the HIV positive-DVT group reflects low systemic iron status, but the raised serum ferritin (although not statistically significant) may be due to the inflammatory status of the individuals.

**Table 2:** Analysis of inflammatory markers using one-way ANOVA with Tukey’s multiple comparison test

Inflammatory marker (normal reference range)	Control mean (std. dev) [range] {% in normal range}	HIV negative-DVT mean (std. dev) [range] {% in normal range}	HIV positive-DVT mean (std. dev) [range] {% in normal range}	P value	Significant difference < 0.05		
					Control vs. HIV negative-DVT	Control vs. HIV positive-DVT	HIV negative-DVT vs. HIV positive-DVT
WCC (M: 3.9–10.4 × 10^9^/L F: 3.9–12.6 × 10^9^/L)	5.3 (0.9) [4.0–6.7] {80.0}	8.3 (2.4) [4.6–13.8] {94.3}	10.0 (7.8) [4.0–33.4] {83.3}	0.028	–	Yes	–
CRP (< 10 mg/L)	2.3 (2.4) [1–8] {100.0}	60.9 (59.1) [1–193] {28.1}	92.3 (76.6) [7–245] {8.3}	0.003	Yes	Yes	–
ESR (0–10 mm/h)	5.0 (4.6) [1–15] {77.8}	32.1 (36.6) [2–139] {46.9}	46.4 (38.3) [9–116] {9.1}	0.041	–	Yes	–
Fibrinogen (2–4 g/L)	2.6 (0.7) [1.7–4.3] {70.0}	3.6 (1.3) [1–7] {67.7}	3.4 (1.2) [1.5–5.3] {63.6}	0.086	–	–	–
Serum iron (M: 11.6–31.3 µmol/L F: 9.0–30.4 µmol/L)	18.0 (9.0) [3–33] {90.0}	8.7 (6.3) [2–29] {23.3}	6.1 (3.6) [1.8–13] {10.0}	0.0002	Yes	Yes	–
Transferrin (M: 2.2–3.7 g/L F: 2.5–3.8 g/L)	3.0 (0.7) [2.3–4.5] {90.0}	2.4 (0.7) [2.3–4.5] {50.0}	1.9 (0.9) [0.6–3.2] {33.3}	0.007	–	Yes	
Iron Saturation (M: 20–50% F: 15–50%)	26.6 (15.2) [3–53] {70.0}	17.7 (19.3) [3–97] {25.9}	20.4 (28.3) [2–88] {12.5}	0.505	–	–	–
Ferritin (µg/L) (M: 26–388 µg/L F: 8–252 µg/L)	147.6 (131.3) [3–412] {80.0}	187.3 (237.6) [1.3–1101] {77.8}	240.4 (159.2) [7–487] {55.6}	0.620	–	–	–

*HIV* human immunodeficiency virus, *vs.* versus, *WCC* white cell count, *CRP* C-reactive protein, *ESR* erythrocyte sedimentation rate

**Table 3:** Analysis of haematological markers using one-way ANOVA with Tukey’s multiple comparison test

Haematological marker (normal reference range)	Control mean (std. dev) [range] {% in normal range}	HIV negative-DVT mean (std. dev) [range] {% in normal range}	HIV positive-DVT mean (std. dev) [range] {% in normal range}	P value	Significant difference < 0.05		
					Control vs. HIV negative-DVT	Control vs. HIV positive-DVT	HIV negative-DVT vs. HIV positive-DVT
RCC (M: 4.2–5.9 × 10^12^/L F: 3.9–5.4 × 10^12^/L)	5.3 (0.4) [4.5–5.8] {90.0}	4.3 (0.9) [2.19–6.98] {65.7}	3.7 (0.7) [2.5–5.0] 33.3}	0.0003	Yes	Yes	–
Hb (M: 13.4–17.5 g/dL F: 11.6–16.4 g/dL)	14.9 (1.5) [12.3–16.4] {100.0}	12.7 (3.2) [4.6–18.5] {71.4}	9.8 (3.3) [4.1–16.7] {25.0}	0.0008	–	Yes	Yes
Hct (M: 0.4–0.5 L/L F: 0.3–0.5 L/L)	0.5 (0.1) [0.4–0.5] {80.0}	0.4 (0.1) [0.2–0.6] {77.1}	0.3 (0.1) [0.2–0.5] {33.3}	0.0004	Yes	Yes	Yes
MCV (M: 83.1–101.6fL F: 78.9–98.5fL)	90.6 (9.4) [74–108.2] {80.0}	89.8 (7.9) [57.7–102.8] {85.7}	84.6 (11.2) [63.1–103.6] {75.0}	0.183	–	–	–
MCH (M: 27.8–34.8 pg F: 26.1–33.5 pg)	28.5 (2.8) [21.6–32.5] {70.0}	29.1 (3.7) [15.3–36] {82.9}	25.7 (5.1) [15.5–33.5] {66.7}	0.047	–	–	Yes
MCHC (M: 33–35 g/dL F: 32.7–34.9 g/dL)	31.5 (1.5) [29.2–33.8] {70.0}	32.2 (2.2) [26.2–35.5] {45.7}	30.3 (3.0) [23.3–33.3] {33.3}	0.038	–	–	Yes
RCDW (M: 12.1–16.3% F: 12.4–17.3%)	14.4 (1.6) [12.6–16.9] {90.0}	15.0 (2.4) [11.9–22.1] {82.9}	16.8 (3.1) [12.1–22.8] {58.3}	0.040	–	–	–
Plt (M: 171–388 × 10^9^/L F: 186–454 × 10^9^/L)	232.9 (35.8) [169–296] {90.0}	287.2 (128.2) [36–7] {71.4}	358.6 (182.5) [149–796] {58.3}	0.087	–	–	–
MPV (M: 7.1–11.0 fL F: 7.3–11.3 fL)	8.978 (1.2) [6.9–10.3] {88.9}	8.8 (1.1) [7.1–11.4] {94.3}	9.0 (0.9) [7.9–10.8] {100.0}	0.779	–	–	–

*Haem.* haematological, *HIV* human immunodeficiency virus, *vs.* versus, *RCC* red cell count, *Hb* haemoglobin, *Hct* haematocrit, *MCV* mean corpuscular volume, *MCH* mean corpuscular haemoglobin, *MCHC* mean corpuscular haemoglobin concentration, *RCDW* red cell distribution width, *Plt* platelet count, *MPV* mean platelet volume

Inflammation is reflected, whether from the DVT or the HIV infection, by the raised CRP and ESR. Surprisingly, the platelet count was not decreased in the HIV positive-DVT group. We expected this parameter, as well as the MPV to be markedly decreased, due to, e.g. HIV thrombocytopaenia, which is usually common amongst HIV patients, but in our sample this was not the case.

### Thromboelastography

Table 4 shows a comparison of the WB and PPP TEG results between the various groups. The WB and PPP, in the HIV negative-DVT and HIV positive-DVT groups, are suggestive of clot hypercoagulability and it is reflected by a rapid R-time, K-time, MRTG and TMRTG. However, with regard to the WB, only the R-time and TMRTG in the HIV-DVT group compared to the control group; and only the TMRTG in the HIV negative-DVT group compared to the control group were statistically significant. The PPP only demonstrated a statistically significant difference with the K-time in both the DVT groups (HIV positive and HIV negative) compared to the control group. Interestingly, there were no significant differences in hypercoagulability between the HIV positive-DVT and the HIV negative-DVT groups.

**Table 4:** TEG results of WB and PPP using one-way ANOVA with Tukey’s multiple comparison test

TEG Parameters (normal reference range)	Control mean (std. dev) [range]	HIV negative-DVT mean (std. dev) [range]	HIV positive-DVT mean (std. dev) [range]	P value	Significant difference < 0.05		
					Control vs. HIV negative-DVT	Control vs. HIV positive-DVT	HIV negative-DVT vs. HIV positive-DVT
Whole blood analysis							
R-time (9–27 min)	8.3 (2.7) [5.2–13.7]	6.3 (1.8) [3.7–12.2]	5.8 (2.9) [2.2–11.8]	0.032	–	Yes	–
K-time (2–9 min)	4.2 (1.1) [3.2–6.9]	3.4 (2.2) [1.3–11.5]	2.7 (1,6) [0.8–6.1]	0.180	–	–	–
Alpha angle (22–58°)	53.7 (4.3) [46.2–59.2]	52.9 (14.24) [17.7–78.1]	58.4 (13.8) [32.1–78.7]	0.437	–	–	–
MA (44–64 mm)	56.1 (6.6) [45–63.5]	55.6 (12.6) [29.4–79.9]	56.4 (12.8) [39.2–80.9]	0.979	–	–	–
MRTG (0–10dcs)	3.4 (1.1) [1.6–4.79]	5.8 (3.4) [1.7–14.3]	6.9 (5.3) [1.9–21.7]	0.074	–	–	–
TMRTG (5–23 min)	12.6 (3.7) [8.3–19.7]	9.1 (3.0) [4.3–20.1]	8.2 (3.7) [3.2–15.7]	0.008	Yes	Yes	–
TTG (251–1014 dcs)	669.3 (168.0) [401.1–876.1]	652.2 (406.7) [101.3–1830]	769.9 (478.2) [322–1999]	0.666	–	–	–
Platelet poor plasma analysis							
R-time (9–27 min)	7.8 (1.1) [6.4–9.5]	7.2 (2.7) [3.2–16.4]	6.0 (1.9) [2.2–8.6]	0.166	–	–	–
K-time (2–9 min)	4.8 (4.1) [1.2–15.0]	2.1 (1.9) [0.8–10.9]	2.0 (1.7) [0.8–6.9]	0.016	Yes	Yes	–
Alpha angle (22–58°)	65.9 (7.2) [53.5–76.6]	63.3 (13.8) [23.2–79.9]	66.5 (14.7) [31.8–82.2]	0.731	–	–	–
MA (44–64 mm)	30.7 (6.5) [21.5–41.0]	37.0 (9.5) [10.1–51.8]	37.9 (16.0) [16.6–70.3]	0.235	–	–	–
MRTG (0–10 dcs)	6.2 (4.0) [2.3–15.3]	9.2 (3.8) [1.3–17.2]	9.6 (5.7) [3.1–23.4]	0.118	–	–	–
TMRTG (5–23 min)	9.4 (1.3) [7.7–11.7]	9.0 (3.8) [4.3–23.0]	7.1 (2.2) [2.8–9.7]	0.164	–	–	–
TTG (251–1014 dcs)	230.8 (69.3) [140.3–347.5]	310.4 (111.5) [56.0–537.5]	363.9 (312.2) [100.0–1193.0]	0.213	–	–	–

*TEG* thromboelastography, *HIV* human immunodeficiency virus, *vs.* versus, *R* reaction, *K* kinetics, *MA* maximum amplitude, *MRTG* maximum rate of thrombus generation, *TMRTG* time to maximum rate of thrombus generation, *TTG* total thrombus generation, *PPP* platelet poor plasma

### Scanning electron microscopy

**Fig. 1:**
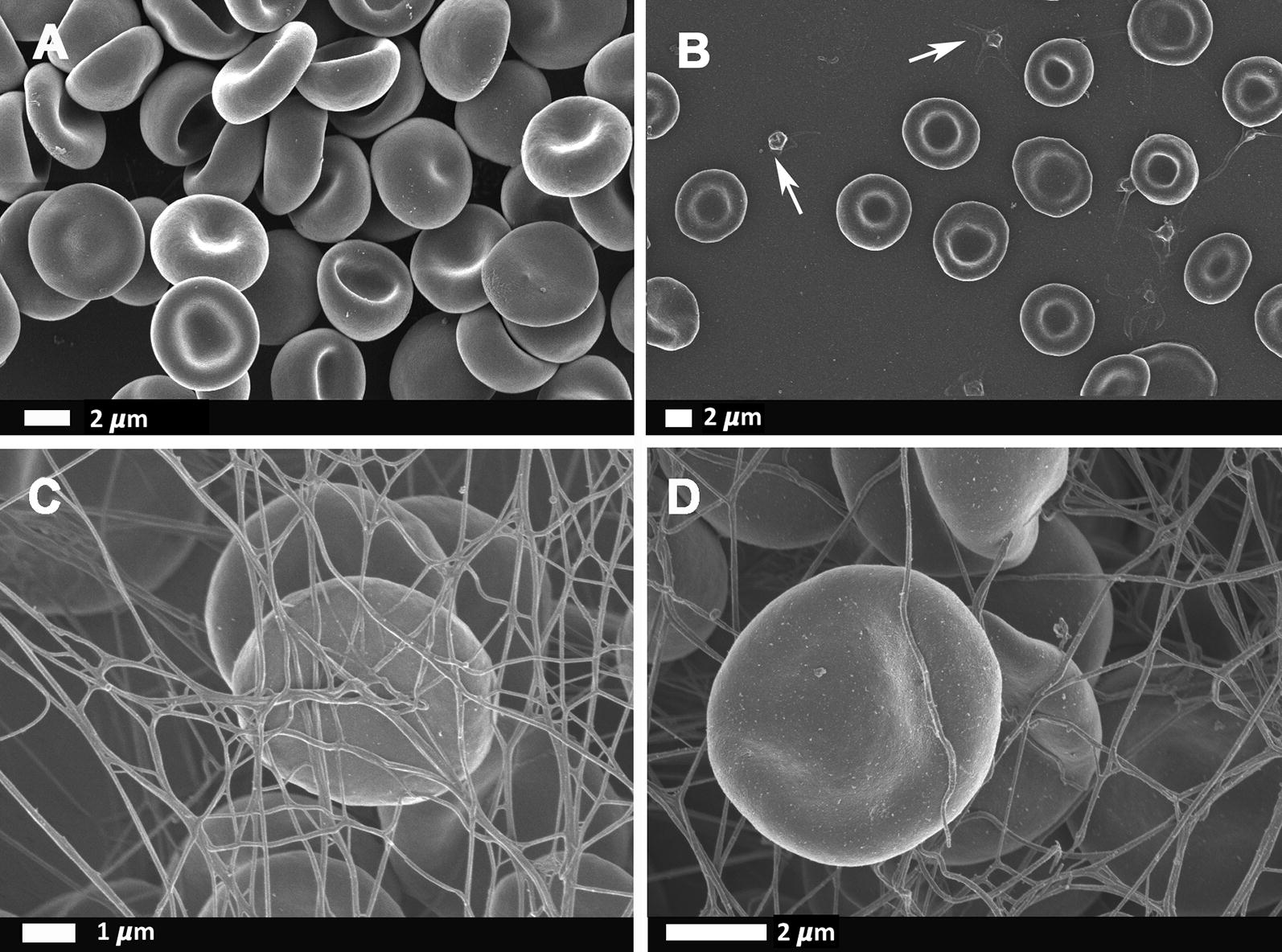
Scanning electron microscopy micrographs of **a** comparison with representative healthy RBCs from other studies [8], **b** representative healthy platelets (see arrows) and RBCs from the current study, **c**, **d** after addition of thrombin to whole blood, where fibrin fibres are formed over the discoid RBCs with no cellular distortion (from the current study)

**Fig. 2:**
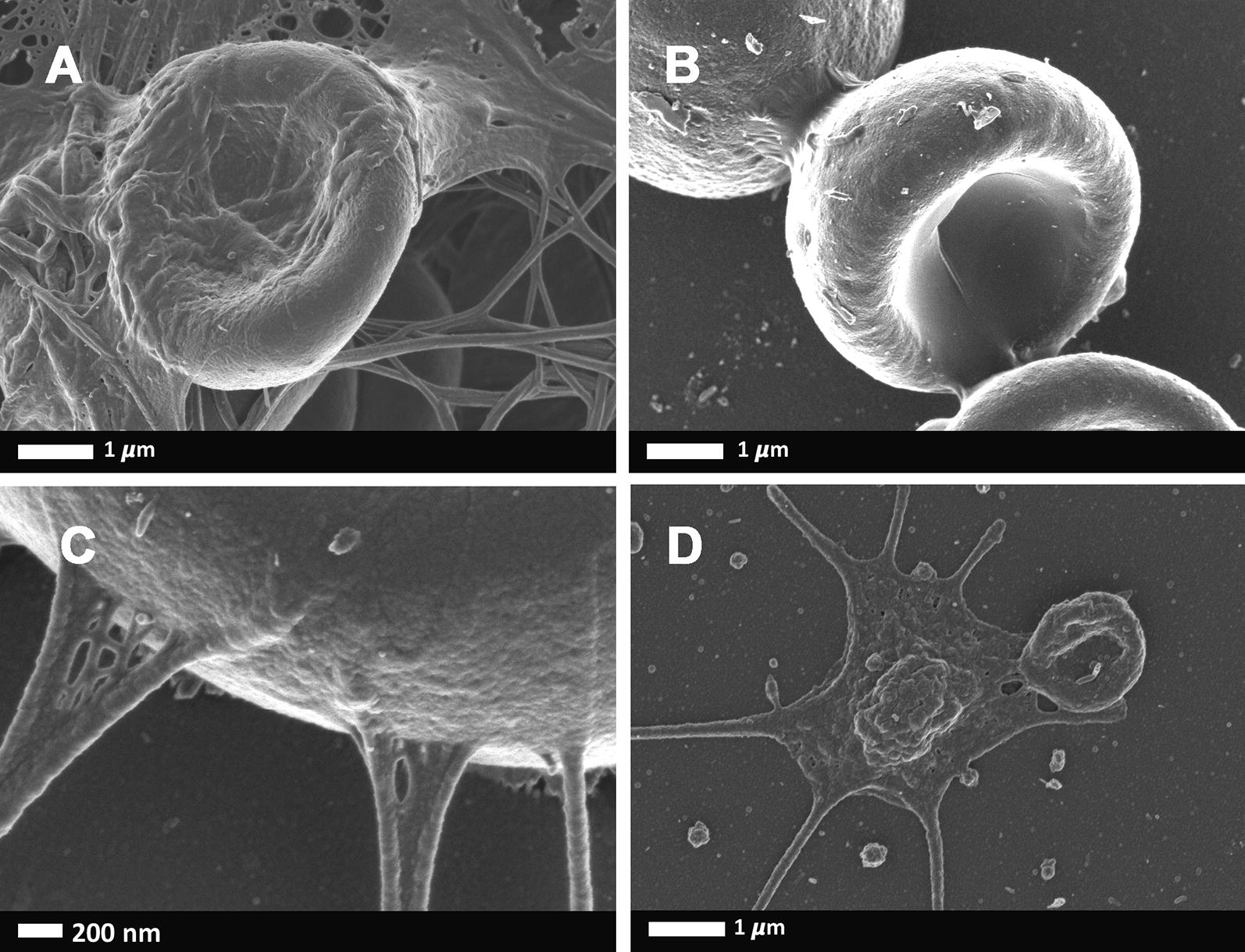
Representative scanning electron microscopy micrographs of RBCs from HIV negative-DVT patients. **a** Whole blood with thrombin, showing RBC entrapped in fibrin matter, **b** RBCs agglutinated to each other (no thrombin), **c** higher magnification showing agglutinated plasma proteins attached to the RBC membrane and **d** a hyperactivated platelet

**Fig. 3:**
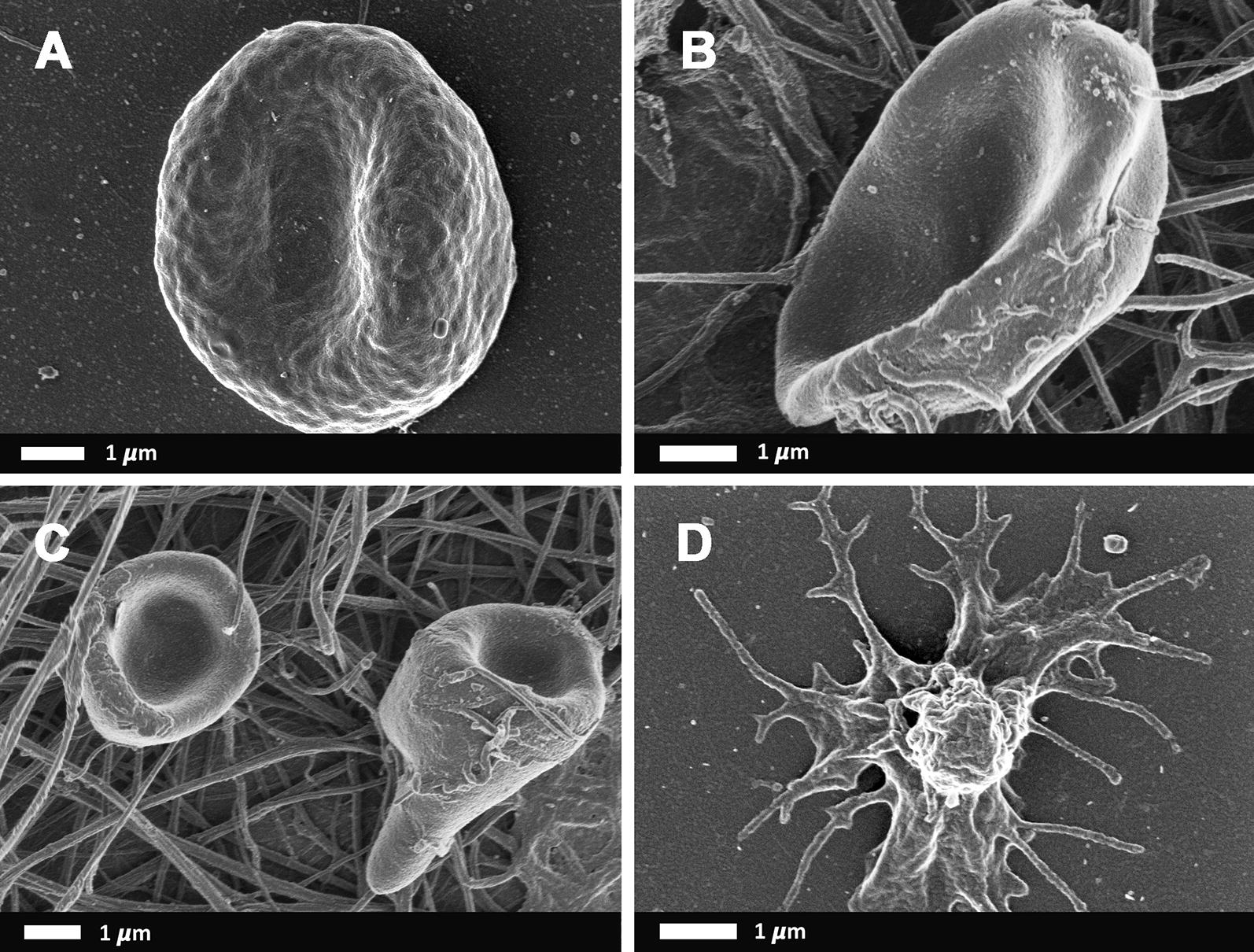
Representative scanning electron microscopy micrographs of RBCs and platelets from HIV positive-DVT patients. **a** RBC with pathological membrane; **b**, **c** whole blood with thrombin, showing RBCs trapped in dense matted fibrin deposits and **d** a hyperactivated platelet

SEM micrographs of representative healthy RBCs and platelets are shown in Fig. 1, while Figs. 2 and 3 show SEM of RBCs and platelets in HIV negative-DVT and HIV positive-DVT patients.

## Discussion

Both DVT groups (HIV negative and HIV positive groups) had parameters suggesting anaemia compared to the control group (Table 3). However, the RBC count, Hb and Hct levels in the HIV negative-DVT group, even though lower than the control group, are still within the normal reference ranges [29]. The Hb and Hct in the HIV positive-DVT group were significantly lower than both the HIV negative-DVT and control groups; and lower than the normal reference ranges indicating an anaemia. Anaemia is commonly found in HIV positive patients but the cause of the anaemia is not always clear [30–35]. An inadequate erythropoietin feedback mechanism is suspected to be a major contributor in HIV-related anaemia [30]. A low reticulocyte count is commonly found with associated polychromasia (abnormally high number of immature RBCs), indicating a possible under-producing bone marrow [30, 36–38]. Other factors that contribute to HIV-associated anaemia, includes intestinal malabsorption, autoimmune haemolysis, bone marrow malignancies, blood loss and opportunistic complications [30, 32, 38, 39]. Even with the decreased RBC count, Hb and Hct levels in the HIV negative-DVT group (as compared to the control group), there were no significant differences with MCV, MCH as well as MCHC (Table 3). The changes in the HIV negative-DVT group may support an anaemia typically associated with inflammation, also known as anaemia of chronic disorders [30, 40].

The RCDW is the coefficient of variation of RBC volume. The higher the value, the more anisocytosis (unequal RBC sizes) present. The RCDW of the HIV positive-DVT group was greater than the control and HIV negative-DVT groups (Table 3). A raised RCDW is commonly associated with a decrease in haemoglobin and MCV concentration; but with a raised CRP, fibrinogen and white cell count [41]. This correlates with the haematological and inflammatory markers found in the HIV positive-DVT group (Tables 2, 3). RCDW is strongly associated with mortality. Patel and colleagues reported the all-cause mortality risk increases by 22% for every 1% increase in RCDW. Furthermore, the physiological association between RCDW and mortality has been hypothesised to be related to the systemic factors involved in inflammatory conditions and oxidative stress which affects erythrocyte maturation and degradation [41–43].

The ESR is the extent in which erythrocytes sediment in 1 h [44]. The ESR in both the HIV negative-DVT and HIV positive-DVT groups were raised compared to the control group (Table 2). In inflammatory conditions the ESR rises as the erythrocytes become sticky and adhere to each other which can be seen as rouleaux formation [45–47].

Fibrinogen, a high molecular weight plasma protein, is a crucial factor in the coagulation pathway (factor I). Increased fibrinogen levels are associated with thrombo-embolic events. Fibrinogen also has a role in inflammation as it tends to adhere to the membrane receptors of cells involved with inflammation. Fibrinogen can adhere to the RBCs, which becomes “heavier” resulting in an increased ESR and blood viscosity [48–55]. The fibrinogen levels were greater (but not statistically significant) in the HIV negative-DVT and HIV positive-DVT groups compared to the control group (Table 2) which correlates with the raised ESR levels seen in both groups. Increase fibrinogen concentration in the inflammatory response can explain the raised fibrinogen concentration in the HIV positive-DVT group which contributed to the DVT. The concurrent use of ARV medication has not been shown to affect the fibrinogen concentration [56]. The HIV negative-DVT group with a raised fibrinogen concentration is either due to the DVT resulting in the inflammatory response (and the raised fibrinogen concentration) or the raised fibrinogen concentration contributing to the DVT.

The WCC in the HIV positive-DVT group was statistically significantly raised compared to the control group (Table 2), although still within the normal reference range. Similar to patients with ischemic strokes, thromboembolism results in an inflammatory reaction with raised leukocyte count and CRP [57, 58]. CRP, like the leukocyte count, is an important indicator of inflammatory conditions [59]. HIV is usually associated with a decreased immune function. The elevated leukocyte count in these HIV positive-DVT patients may be due to the HIV infection itself or to opportunistic infections, regardless of whether the patient has a DVT or not. Also, with the ARV treatment HIV is suppressed and the lymphocytes, particularly the CD4 lymphocytes, increase in turnover [60–62]. The concentration of CRP is increased by proinflammatory cytokines, interleukin 1 and 6 [63]. The HIV negative-DVT group has an inflammatory response to the DVT which is reflected by the statistically significantly raised CRP levels compared to the control group (Table 2). The same argument can be made for the raised CRP in the HIV positive-DVT group, however the CRP concentration (as well as fibrinogen) is commonly raised in HIV positive patients compared to the general population even without a DVT [64–68]. The raised CRP in HIV positive-DVT patients (Table 2) indicates a sustained acute phase response [66]. This was statistically significant in the HIV positive-DVT group compared to the control group. The CRP in the HIV positive-DVT group was almost double compared to the HIV negative-DVT group. Increasing CRP concentrations has been reported with HIV disease progression, and this increase does not appear to be affected by ARV treatment [63]. Previously it was noted that increased levels of CRP and fibrinogen are independently highly predictive of 5 year mortality risk in HIV positive patients, especially where the CD4 count is low [69, 70].

Considering all the inflammatory markers (WCC, fibrinogen, CRP and ESR), each marker was statistically significantly raised in the HIV positive-DVT group compared to the control group, with the exception of fibrinogen (Table 2). In the HIV negative-DVT group compared to the control group, only CRP was statistically significantly raised. CRP may therefore be a more sensitive acute phase marker to differentiate an inflammatory condition between DVT patients (HIV negative and HIV positive) compared to healthy subjects. Interestingly, no inflammatory marker was statistically significantly raised in the HIV positive-DVT group compared to the HIV negative-DVT group.

The transferrin, serum iron and iron saturation levels reflects the amount of iron in the body. Transferrin is a plasma protein that transports iron in the blood [62], whereas ferritin is an intracellular structure capable of storing iron atoms. The concentration of serum ferritin is related to the reticuloendothelial iron stores [71]. Serum ferritin and iron concentrations are also indicators for acute phase responses to inflammation [71], although serum ferritin appears to be a better marker of inflammation than iron status [72].

Iron deficiency may be a contributor to anaemia in the HIV positive-DVT group in keeping with a low MCH and MCHC, although this is not reflected with the MCV which was within the normal reference range (Table 3) [73, 74]. A low serum iron and transferrin level seen in the HIV positive-DVT group, but with a raised ferritin level (as compared to the control group), can be explained by an immunologically altered iron metabolism where the body has adequate or increased iron stores but is unable to utilize those stores [32, 37, 60, 61, 71, 75–77]. This functional iron deficiency can be considered a host defence mechanism by withholding iron from possible pathogens [78, 79]. However, as iron is required for normal immune function, iron deficiency can also increase the risk of infection [79].

Although the inflammatory RBC changes have been documented in non-communicable diseases, there are only a few reports of communicable diseases, specifically HIV, and the effect on RBCs and the coagulation system [80–85]. Multiple abnormal RBC shape changes and membrane abnormalities were noted in the patients with DVT (HIV negative and HIV positive groups) (Fig. 1 to 3). During inflammatory diseases, RBCs exposed to oxidative stress and inflammatory molecules undergoes biochemical membrane changes which can result in biophysical shape changes and eryptotic cells [28, 86–93]. Eryptosis is a co-ordinated suicidal death of the red blood cells, similar to apoptosis, that allows for the removal of defective, infected or potentially harmful cells before they undergo haemolysis [94–98]. The abnormal RBCs present with an abnormal expression of phosphatidylserine, a cell membrane lipid, on the external membrane layer. RBCs that display phosphatidylserine also contribute to the hypercoagulation state and they provide a prothrombotic surface for the formation of thrombin [40, 96, 99–110]. Membrane vesicle formation and microparticle shedding (microscopic extracellular membranous structures) were also seen in both DVT groups. RBC-derived microvesicles or microparticles, is known to be associated with the expression of phosphatidylserine [111]. RBC-derived microparticles appear to enhance thrombin generation resulting in a hypercoagulable state, such as in post transfusion DVT, sickle cell disease and haemolytic anaemia [112, 113]. As the microparticle presence might also be associated with increased thrombin presence, the complement system can therefore also be activated and thereby enhance the systemic inflammatory response which is also a hypercoagulable state [114]. Microparticles are also thought to originate from CD4 lymphocytes [115]. As the HIV virus infects CD4 lymphocytes, HIV positive patients may be more prone to developing microparticles and therefore enhancing the hypercoagulable state.

Whole blood with thrombin SEM analysis showed the incorporation of RBCs into the fibrin network. The incorporation of RBCs into the fibrin network stabilises and strengthens the clot by decreasing the permeability of fibrin and increasing the resistance to fibrinolysis [4, 116, 117]. Healthy (discoid) RBCs in netted fibrin fibers are shown Fig. 1c, d. However, in our HIV negative-DVT and HIV positive-DVT groups, the RBCs are trapped in a matted fibrin fiber network. During inflammation, fibrin fibres tend to increase in diameter and assume a matted rather than a netted appearance; while their viscoelasticity may also be influenced by the RBC inclusion in the fibrin network [99]. Also, under conditions of low partial pressure of oxygen, acidosis and in response to mechanical deformation, RBCs release ATP and ADP activating platelets and promoting aggregation and release of platelet granules [113]. This can happen as part of the HIV and DVT pathology. The (hyper) activation of platelets, together with an abnormal matted fibrin network, contracts the clot containing the trapped pathological RBCs into a tight package (Fig. 3b, c). The result is the formation of polyhedrocytes, which is commonly found in DVT [118].

Platelet functioning depends on the quality and the quantity of the platelets [119]. Platelet count is a measure of the number of platelets in a volume of blood. Thrombocytopenia (low platelet count) is a common finding in HIV positive patients, whether it be due to increased destruction or decrease production of platelet cells [56]. However, in this study both the HIV negative-DVT and HIV positive-DVT groups had a non-statistically significant increase in the platelet count (Table 3). It should be noted that platelet count is not always associated with an increased risk of DVT [120]. The mean platelet volume measures the average size of platelets in the blood and is a common platelet activation marker [120–127]. An elevated MPV is associated with low-grade inflammation as well as thrombosis [128]. However, both HIV negative-DVT and HIV positive-DVT groups had a decrease in the mean platelet volume compared to the control group (Table 3). These results may be in keeping with a venous thrombosis where the thrombus is due to activation of the coagulation cascade instead of platelets [129]. It should also be kept in mind that platelets shape and volume do vary, resulting in changes in MPV, even in healthy persons [127]. Together with these results, the ultrastructure of platelets in the HIV positive-DVT group also have features different to that of the control group and the HIV negative-DVT group (Figs. 1b, d, 3d). The control group and the HIV negative-DVT group have (hyper) activated platelet aggregates with smooth intact membranes, pseudopodia formation, openings of the open canalicular channels and membrane blebbing interspersed among smooth intact membranes. These are the typical morphological features of activated platelets seen in healthy individuals [130]. The HIV positive-DVT patients have activated platelet aggregates which are also seen to have the same features; but with the addition of shrivelled aggregates with irregular membranes, torn membrane surface and shedding of procoagulant vesicles. These features are suggestive of apoptosis, cell death, as was seen in the red blood cells. Similar ultrastructural changes in HIV patients were documented by Pretorius et al. in 2008 [80]. These ultrastructural changes may be due to altered viral infected megakaryocyte morphology or due to direct infection and damage by the HIV virus. The HIV virus may gain entry into the platelets by undergoing phagocytosis or through the openings of the open canalicular system channels [80, 131, 132]. Regardless of the way of entry, platelets containing the HIV virus are activated. It is not clear if the platelets containing the virus facilitates viral replication and spreading; or assists in clearance of the virus [131]. With the latter, the ultrastructural changes may be due to the immune response of the body resulting in antibody-induced destruction of the platelets [80]. Antiretroviral medication has been shown to have platelet related effects such as a decreased prevalence of HIV associated thrombocytopaenia but may have an increased bleeding risk [133]. This increased bleeding risk may be explained by the ultrastructural changes seen on the SEM. It is possible that the use of different ARV combinations may result in different morphological changes observed, however to standardize the results, only patients on the primary regimen were recruited for this study.

Our TEG analysis compared well and supported our ultrastructural results (Table 4). The HIV negative-DVT group compared to the control group showed significant differences with regards to TMRTG, while the HIV positive-DVT group compared to the control group, showed significant differences with regards to R-time, and the TMRTG. According to Pretorius and colleagues not all the parameters need be abnormal to indicate pathological coagulability and the degree of coagulability can be related to the number of parameters that are abnormal [5].

Both DVT groups (HIV negative and HIV positive), using whole blood, indicate a hypercoagulable profile that has a rapid initiation and amplification, resulting in the rapid formation of thrombin. The TEG parameters in the HIV positive-DVT group compared to the HIV negative-DVT group indicate a hypercoagulable profile but there were no statistical significance in any of the parameters. Both the HIV negative-DVT and HIV positive-DVT groups have rapid R-times and K-times (Table 4) and trapped RBCs between a matted (hypercoagulable) fibrin network were noted. Considering that pulmonary embolism can result in up to 10% mortality [134] and half of the patients with DVT may have non-symptomatic (silent) pulmonary embolism [135], the changes found on the TEG and SEM may provide for a risk of assessment of the DVT complicating to pulmonary embolism. [136].

## Conclusion

Although there were trends that HIV-positive patients were more hypercoagulable on functional tests (viscoelastic profile) compared to HIV-negative patients, there were no significant differences between the 2 groups. The sample size was, however, small in number. Morphologically there were inflammatory changes in patients with DVT. These ultrastructural changes, specifically with regard to platelets, appear more pronounced in HIV-positive patients which may contribute to an increased risk for hypercoagulability and deep vein thrombosis.

## Data Availability

The dataset(s) supporting the conclusions of this article are available from the authors.

## Abbreviations

ARV: Antiretroviral therapy
AIDS: Acquired immune deficiency syndrome
CRP: C-reactive protein
DVT: Deep vein thrombosis
ESR: Erythrocyte sedimentation rate
Haem: Haematological
Hb: Haemoglobin
HIV: Human immunodeficiency virus
IL: Interleukin
K-time: Kinetic time
MA: Maximum amplitude
MCH: Mean corpuscular haemoglobin
MCHC: Mean corpuscular haemoglobin concentration
MCV: Mean corpuscular volume
MPV: Mean platelet volume
MRTG: Maximum rate of thrombus generation
Plt: Platelet count
PPP: Platelet poor plasma
RBCs: Red blood cells
RCC: Red cell count
RCDW: Red cell distribution width
R-time: Reaction time
SEM: Scanning electron microscopy
TEG: Thromboelastography
TMRTG: Time to maximum rate of thrombus generation
TNF: Tumor necrosis factor
TTG: Total thrombus generation
UNAIDS: Joint United Nations Programme on HIV/Acquired Immune Deficiency Syndrome
WB: Whole blood
WCC: White cell count
